# Neutrosophic goal programming technique with bio inspired algorithms for crop land allocation problem

**DOI:** 10.1038/s41598-024-69487-0

**Published:** 2024-09-16

**Authors:** S. Angammal, G. Hannah Grace

**Affiliations:** School of Advanced Sciences, Department of Mathematics, Vellore Institute of Technology Chennai, Chennai, 6000127 India

**Keywords:** Engineering, Mathematics and computing

## Abstract

In agriculture, crop planning and land distribution have been important research subjects. The distribution of land involves several multi-functional tasks, such as maximizing output and profit and minimizing costs. These functions are influenced by a variety of uncertain elements, including yield, crop price, and indeterminate factors like seed growth and suitable fertilizer. In order to address this problem, other researchers have used fuzzy and intuitionistic fuzzy optimization approaches, which did not include the indeterminacy membership functions. However, the neutrosophic optimization technique addresses the problem by using individual truth, falsity, and indeterminacy membership functions. So, to improve the optimal solution, the Neutrosophic Goal Programming (NGP) problem with hexagonal intuitionistic parameters is employed in this study. The membership functions for truth, indeterminacy, and falsity are constructed using hyperbolic, exponential, and linear membership functions. Minimizing the under deviations of truth, over deviations of indeterminacy, and falsity yields the NGP achievement function, which is used to attain optimal expenditure, production, and profit under the constraints of labour, land, food requirements, and water. Bio-inspired computing has been a major research topic in recent years. Optimization is mostly accomplished through the use of bio-inspired algorithms, which draw inspiration from natural behaviour. Bio-inspired algorithms are highly efficient in exploring large solution spaces, and helps to manage trade-offs between various goals, and providing the global optimal solution. Consequently, bio-inspired algorithms such as Grey Wolf Optimization (GWO), Social Group Optimization (SGO), and Particle Swarm Optimization (PSO) are employed in the current work to determine the global optimal solutions for the NGP achievement function. The data for the study was collected from the medium-sized farmers in Ariyalur District, Tamil Nadu, India. To illustrate the uniqueness and application of the developed method, the optimal solutions of the suggested method are compared with Zimmermann, Angelov, and Torabi techniques. The proposed technique demonstrates that the bioinspired algorithms’ optimal solution to the neutrosophic goal is superior to the existing approaches.

## Introduction

Agriculture is the main and most prevalent industry in Tamil Nadu. In Tamil Nadu, almost 60% of people work as farmers. The agriculture industry provides food for both human and livestock populations. Agriculture provides raw ingredients to a wide range of industries. The current study addresses how to allot crop land to each crop within constraints to provide medium-sized farmers in the Ariyalur area with the maximum return. This district’s key agricultural objectives are to preserve the cropping area, increase productivity and production, double the output of the farming community, and triple the income. Agriculture, which is reliant on the availability of land and water resources, is essential to the nation’s overall development. Planting the right amount of different crops on the right amount of land with enough water for irrigation at the right time is the key to agricultural productivity. Normally, for making decisions in a variety of fields, including public transit, commercial and public industrial activities, and other resource allocation issues, such as agricultural land usage, Linear Programming (LP) is a widely used optimization technique^1,2^. In the field of agriculture, there is uncertainty surrounding yield, industries, technology, and commodity prices. Crisp optimization techniques are not appropriate for crop planning problems because of these unknown aspects. Thus, many academics apply fuzzy multi-objective optimization techniques—introduced by Bellman and Zadeh^3^—to land allocation problems. Just the degree of belongingness is contained in the fuzzy set. By raising the level of objective acceptance, fuzzy programming improves decision-maker satisfaction. It’s important to remember that when figuring out the optimal solution for the Multi-Objective Optimization Problem (MOOP), membership and non-membership functions are equally important. Thus, Attanasov^4^ introduced the intuitionistic fuzzy set, which is an extension of the fuzzy set. Numerous decision-making situations call for the application of intuitionistic and fuzzy optimization strategies. Even though fuzzy sets and intuitionistic fuzzy sets have made significant progress in handling various forms of uncertainty, a contemporary paradigm is still required to address issues such as indeterminacy. Consequently, Smarandache^5^ developed the Neutrosophic Set to cope with related issues pertaining to indeterminacy, rejection, and acceptance. Using the neutrosophic set, Surapati Pramanik et al.^6^ proposed neutrosophic number goal programming for solving multi-objective LP problems. This method can be used in production planning and medical management. Mohammad Fallah et al.^7^ introduced the neutrosophic programming approach to construct a multi-objective sustainable biomass supply chain network with optimal overall costs, employment, greenhouse gas emissions, and product transfer time. Compared to classical optimization techniques, the field of neutrosophic optimization is quite recent. Not every sort of optimization problem is a good fit for neutrosophic optimization. It might only apply to particular fields or kinds of issues where ambiguity, vagueness, or indeterminacy are major factors. In order to efficiently search the space and find the global optimal solutions to optimization problems, bio-heuristic algorithms are designed. It does not require derivative knowledge about the objective function, in contrast to gradient-based optimization approaches. This makes them appropriate for optimization situations in which evaluating the objective function is computationally costly, discontinuous, or non-differentiable. Bio-heuristic algorithms balance exploration and exploitation. Because of this balance, they are able to efficiently traverse intricate search spaces, avoiding early convergence to inferior solutions and making effective use of potential search spaces. Consequently, certain bio-inspired algorithms are employed in this study to determine the global optimal solution for the NGP.

Nowadays, bio-inspired algorithms play a phenomenal role in solving many real-life application problems. Bio-inspired optimization algorithms are techniques for effectively resolving optimization problems in a wide range of application domains that are typically motivated by specific characteristics of living organisms, physical laws, and theories of evolution. Particle Swarm Optimization (PSO), Cuckoo Search Optimization (CSO), Grey Wolf Optimization (GWO), Ant Colony Optimization (ACO), Social Group Optimization (SGO), and Whale Optimization Algorithm (WOA) are a few examples of bio-inspired optimization techniques that have attained popularity in recent times for their ability to optimize solutions in fields like biology, computer science, and mathematics. Ejieji and Akinsunmade^8^ integrated flower pollination and dragonfly algorithms and applied them for optimal crop field allocation in order to maximize net production. Animak Naik et al.^9^ used a social group optimization (SGO) algorithm that was motivated by human social behaviour to solve multi-modal functions and data clustering challenges. GWO, which mimics the social hierarchy and hunting technique of grey wolves, has been used by Shahverdi and Maestre^10^ and coupled with Irrigation Canal System Simulation (ICSS) to plan water supply. To analyse the importance of water allocation in increasing yield production, Hossein Behdarvandi^11^ employed the GWO technique for optimal water allocation. Thilagavathi et al.^12^ employed the LINGO solver, the social spider algorithm, and the ant colony optimization algorithm to solve crop planning problems in order to optimize net profit while using the least amount of water. Farshad Rezaei et al.^13^ employed a multi-objective PSO algorithm with a fuzzy basis for enhancing groundwater resource sustainability, minimizing shortages in irrigation needs, and maximizing agricultural net benefits. Using GA, PSO, and CSO, Gouri Sajith et al.^14^ presented a combinatorial optimization strategy for land allocation that takes into account environmental, agronomic, hydro-climatic, and socio-economic objectives, and they also found that GA was a more effective solution than PSO and CSO. Beatriz Flamia Azevedo et al.^15^ employed PSO, NSGA-II, and GWO to solve the production scheduling problem. Also, an automatic bio-inspired clustering algorithm based on the GA analyses the final pareto front to choose the best optimal solution. Combining the convergence skills of SGO with the exploratory powers of WOA in a perfectly balanced exploration-exploitation ratio, Kalananda and Komanapalli^16^ created two hybrid optimization algorithms such as HS-WOA and HS-WOA+. Applications for these hybrid optimization approaches include data classification, vehicle routing, production scheduling, and other related activities. A Multi-Objective Marine Predator Algorithm (MOMPA) was proposed by Pradeep Jangir et al.^17^ to solve the MOOP. Additionally, the suggested method was evaluated against the Water-Cycle Algorithm, Symbiotic-Organism Search, and Moth-Flame Optimizer algorithms to confirm its superiority. The Liver Cancer Algorithm (LCA), developed by Essam Houssein^18^, is a novel bio-inspired optimization algorithm that replicates the growth and takeover process of liver tumours. It was created to effectively balance local and global searches and explore the search space by utilizing genetic operators in conjunction with a Random Opposition-Based Learning (ROBL) approach. It is useful for resolving feature selection and mathematical benchmark issues.

Researchers have recently focused a great deal of attention on the Slime Mould Algorithm (SMA), which was developed by Shimin Li et al.^19^ based on the oscillation mode of slime mould. This is because of the algorithm’s excellent optimization capabilities, straightforward structure, and acceptable convergence in resolving a wide range of challenging real-world problems. Bhandakkar and Mathew^20^ combine WOA and SMA to provide the best possible allocation of hybrid power flow controllers in power systems. Mohamed Abdel-Basset et al.^21^ also integrated SMA with WOA to maximize the entropy in order to solve the picture segmentation issue for COVID-19 chest X-ray images. For the purpose of optimizing steel frame structures, Gholizadeh et al.^22^ used the Moth-Flame Optimization (MFO) algorithm, which was created by Mirjalili et al.^23^ and is based on the navigation technique of natural moths. Xuefeng Dai and Yang Wei^24^ suggested an enhanced MFO technique to determine the mobile robot’s global optimal path. Hunger Games Search (HGS) is one of the metaheuristic algorithms that mimics an animal’s foraging and hunger instincts. For sustainable agricultural and food supply chain management strategies, Zheng Xu et al.^25^ created a Hunger Games Search (HGS) optimization using a deep learning model in order to maximize income. Ali Asghar Heidari et al.^26^ bring forward a novel population-based, nature-inspired optimization methodology known as the Harris Hawks Optimizer (HHO). Harris’s hawks’ cooperative nature and chasing technique serve as the primary source of inspiration for HHO. HHO is a relatively new method that has drawn the interest of numerous academics for its ability to tackle a variety of optimization issues, including single- and multi-objective, unconstrained, constrained, continuous, discrete, linear, nonlinear, and mixed-integer nonlinear problems^27–30^. Hang Su et al.^31^ have suggested a novel high-performance optimization technique that relies on the rime generation process. This approach effectively addresses intricate optimization issues, including feature selection and image segmentation. Using the aforementioned metaheuristic techniques, globally optimal solutions are developed for a wide range of real-world problems. Therefore, in this study, bio-inspired algorithms such as PSO, GWO, and SGO are used to discover the global optimal solution for the achievement function created for the optimal crop land allocation problem.

### Motivation and contribution

Numerous academics have employed intuitionistic and fuzzy optimization techniques, which essentially take truth and falsity membership functions into account, to address the crop land allocation problem. However, several unpredictability can occur in agriculture, such as crop development, seed growth, and fertilizer use. In crop planning problems, fuzzy and intuitionistic fuzzy sets capture all types of uncertainty, but a modern framework is needed to explain indeterminate comprehension. Thus, the neutrosophic approach is a superior optimization technique to identify the best solution for an ambiguous and indeterminate problem. All of these are the motivations behind creating a new NGP achievement function to maximize farmer profitability. The following are the primary goals and contributions of the current study:

- To optimize the production, expenditure, and profit of a medium-sized farm owner in Ariyalur district under the constraints of food requirements, labour, water, and land.
- Creating a new NGP problem using linear and non-linear membership functions and using PSO, GWO, and SGO for obtaining the global optimal solution for the NGP achievement function.

India is the most populous nation, and its daily food needs are growing. The Indian government has launched several new programmes in an effort to meet the nation’s rising food demand. Crop planning is essential for agriculture to control the demand for food. An efficient strategy for allocating crop land can control seasonality, soil fertility, and variability in productivity. The suggested method helps manage the uncertainty and indeterminacy of crop planning and finds the most acceptable solution by fusing bio-inspired algorithms with a neutrosophic goal. The proposed technique can also be used to find the optimal solution for the numerous real-world MOOPs with indeterminacy and unclear aspiration levels.

## Literature review

Optimizing return with minimal expenditure on restricted land is a primary goal in agriculture. To boost crop yield, Meselu Tegenie et al.^32^ recommended linear programming—based crop land allocation for small farm holders. Mahak Bhatia et al.^33^ employed a linear programming approach to determine the best crop combination for the large farm owner in Jaipur in order to increase productivity. Since most farm planning issues are multi-objective in nature and LP is a single-objective optimization technique, the goal programming (GP) technique, one of the popular tools for multi-objective decision analysis, is used to solve the problem of land allocation planning for the optimal production of a variety of crops. To increase farmers production and returns, Elizabeth Gosling et al.^34^ evaluated agroforestry systems using a goal-programming approach. Animesh Biswas et al.^35^ and Sharma et al.^36^ proposed pre-emptive and weighted fuzzy goal programming techniques for land use planning to improve the yield of farmers. Srivastava et al.^37^ proposed a two-level fuzzy goal programming technique for the land allocation problem in the canal command area. For the sustainable crop pattern, Ramtin Joolaie et al.^38^ proposed a fuzzy goal programming method with a random sampling approach. For the production distribution with minimum transportation cost and delivery time Srikant Gupta et al.^39^ proposed efficient fuzzy goal programming which is the combination of goal program, fuzzy program and interactive program. Demmelash Mollalign Moges et al.^40^ proposed a two-phase weighted intuitionistic fuzzy goal programming technique to solve an intuitionistic fuzzy multi-objective linear fractional optimization problem, and for the efficiency of the algorithm, the author applied it to an agricultural production planning problem. Angammal et al.^41^ compared fuzzy and intuitionistic fuzzy optimization methods for three-season’s crop land allocation problem. Bharati et al.^42^ developed intuitionistic fuzzy optimization using fractional programming to maximize small farm holders profits and production. Intuitionistic fuzzy optimization was developed by Pawar et al.^43^ to maximize net profit and employment generation with minimal cultivation cost.

Real-life complications can lead to indecision or neutral thinking when making the best decisions. The degree of indeterminacy is also important in decision-making, along with membership and non-membership. Thus, Sajida Kousar et al.^44^ offered neutrosophic optimization to maximize rice and wheat productivity and profit in Pakistan’s kharif and rabi seasons. To minimize production and inventory holding costs and maximize net profit for the hardware firm’s multi-product manufacturing problem, Mohammad Faizal Khan et al.^45^ developed neutrosophic and intuitionistic fuzzy optimization. Angammal et al.^46^ proposed an INPA with exponential and linear membership functions to optimize land allocation for medium-sized farm owners. Indrani Maiti et al.^47^ proposed goal programming with interval parameters for the multi-level, multi-objective linear programming problem with the neutrosophic number parameter. Firoz Ahmad et al.^48^ addressed the multi-level, multi-objective fractional programming problem with a rough interval parameter using neutrosophic goal programming. A unique neutrosophic programming approach was proposed by Sapan Kumar Das et al.^49^ to solve the linear programming problem with pentagonal neutrosophic number parameter. Suizhi Luo et al.^50^ proposed three interactive programming approaches to solve the multi-level programming problem in the neutrosophic environment, including the Score function based interactive approach, the TOPSIS based interactive approach, and the Multi Objective Optimization on the basis of Ratio Analysis (MOORA) based interactive approach. Firoz Ahmad et al.^51^ proposed an interactive neutrosophic programming method to manage energy, food, and water security. Complex optimization problems can be solved with high-quality solutions using bio-inspired optimization methods. Therefore, Xiaoping Liu et al.^52^ presented hybrid PSO, a new particle adjustment rule with genetic reproduction mechanisms, crossover and mutation with system dynamics for land distribution in Panyu, Guangdong, China. A modified social group optimization technique was presented by Swagato Das et al.^53^ for damage assessments of modelled civil engineering structures. To maximize net benefits and minimize fertility usage Crow Search Algorithm (CSA) and PSO were combined by Sonal Jain et al.^54^ Ashutosh Rath et al.^55^ used swarm intelligence techniques including GA, cuckoo search (CS), and PSO to create a cropping pattern that maximized net return for the Hirakud command region in India. Zhidong Wang et al.^56^ used GWO to find the pareto optimal solution for agricultural water allocation in Aksu Valley, Xinjiang, China. To predict the accurate fertilizer application ratio and improve the yield Cengcheng Chen et al.^57^ proposed the novel multi strategy GWO algorithm. Alireza Donyaii et al.^58^ proposed the multi-objective GWO algorithm to minimize vulnerability and maximize the reliability of the optimum rules for operating Golestan Dam, located in Iran, under climate change conditions. From the above literature review, it is clear that various agricultural problems have been solved by using LP, GP, fuzzy, intuitionistic fuzzy, neutrosophic approach, and bioinspired algorithms. But for crop land allocation problems, which involve many uncertainty and indeterminacy factors, the NGP with linear and non-linear membership functions is not yet used. Thus, for determining the most suitable land allocation for each crop, the neutrosophic goal programming approach is offered here, and also PSO, SGO, and GWO employed in this study to get the global optimal solution for the achievement function.

## Study area

**Figure 1:**
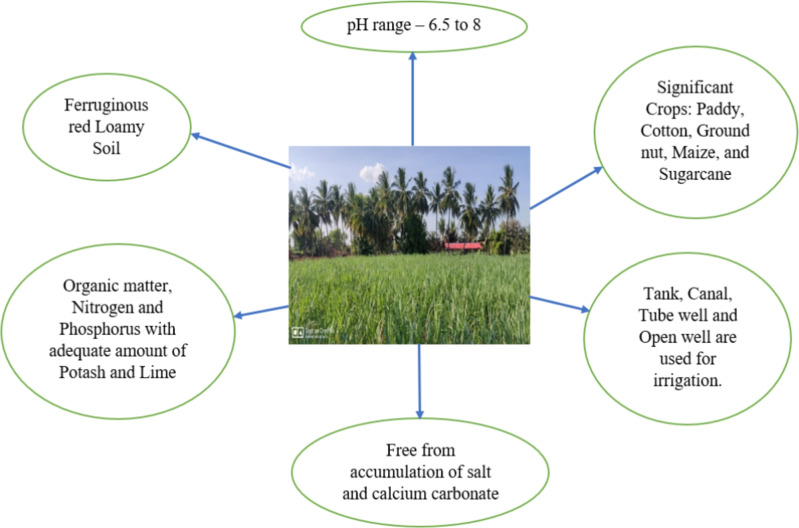
Soil, irrigation and crops cultivation in Ariyalur district.

The district of Ariyalur, which has excellent agricultural practices, provided all of the agricultural data needed for this study. The Ariyalur District covers a land area of 1,93,338 hectares. 1,11,874 hectares are under cultivation. There are approximately 66,738 hectares of rain-fed land and 45,136 hectares of irrigated land. The majority of inhabitants in this district work mostly in agriculture, where they cultivate a wide range of crops. 1% of farmers in the district of Ariyalur are medium-sized farmers. The Ariyalur district agricultural land type, irrigation systems, and major cultivated crops are presented in Fig. 1. The medium farmer in Narasingapuram village, Ariyalur district, Tamil Nadu, provided the cultivation data for the present study. In that district, the 15-acre farmer cultivated paddy, cotton, and groundnut in the kharif; paddy, sweet corn, groundnut, and pearl millet in the rabi; and paddy, brinjal, sesame, and onion in the summer season. This research optimizes productivity, profit, and other costs, including labour, seed, fertilizer, pesticides, and miscellaneous charges within land, water, labour, and food restrictions. The methods utilized to identify the best solution and to produce the neutrosophic achievement function will be covered in the following section.

## Methodology

**Figure 2:**
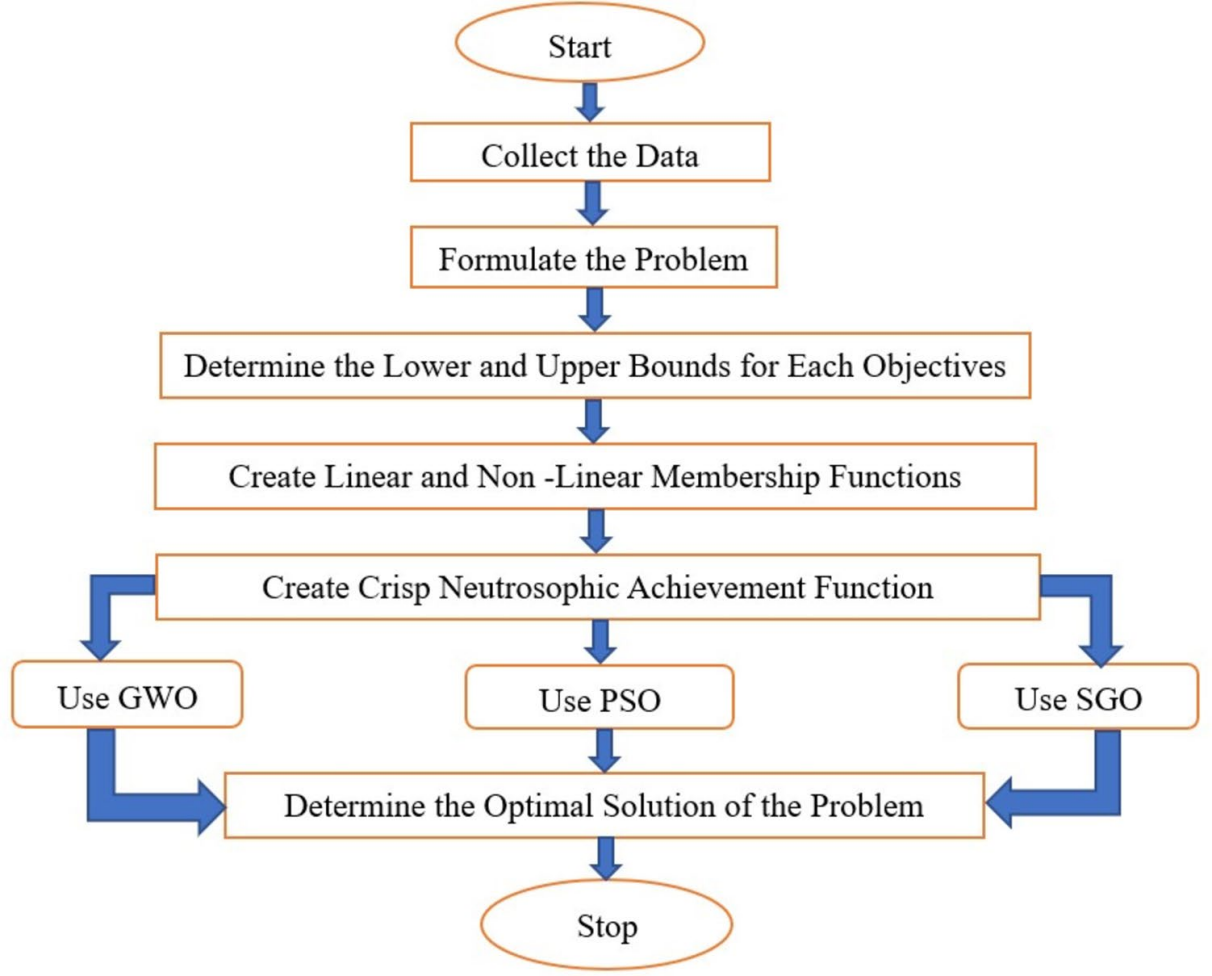
Flowchart of neutrosophic goal with bio inspired algorithm.

Among all the methods discussed in the literature review, no one has developed a neutrosophic goal programming technique for crop land allocation problems that includes linear, hyperbolic, and exponential membership functions. Instead, many researchers employed intuitionistic fuzzy and fuzzy goal programming with linear membership functions and linear goal programming strategies for multi-objective crop land allocation problems. As a result, this paper’s methodology attempts to address this problem. The problem of allocating agricultural land has intuitionistic parameters. Therefore, the author begins by estimating the intuitionistic parameter’s value. After evaluation, the crisp neutrosophic objective function is produced by minimizing the over deviation of untruth and indeterminacy and the under deviation of truth. Bio-inspired optimization algorithms use nature’s biological evolution to create robust solutions. Therefore, the crisp neutrosophic goal programming problem is solved by using some bio-inspired algorithms named PSO, GWO, and SGO. Although these three algorithms are frequently employed in real-world scenarios, they have not been utilised in the context of the neutrosophic goal programming technique to address the crop land allocation problem. The proposed methodology’s process is depicted in Fig. 2.

### Particle Swarm Optimization (PSO)

In 1995, James Kennedy and Russell C. Eberhart^59^ created PSO, a stochastic optimization method based on swarm intelligence. PSO solves problems through social interaction. A swarm of particles searches the search space for the optimal solution. Each particle’s velocity is dynamically changed by its movement experience and that of its neighbours. The global best $(gbest_{pi})$ is the particle with the highest fitness value, while the personal best $(pbest_{pi})$ is the particle’s best position. The PSO algorithm optimizes by following $(pbest_{pi})$ and $(gbest_{pi})$. All particles are updated at each step by the following equations.

$$\begin{aligned} v_{pi}(t+1)= & {} w. v_{pi}(t) + c_1. r_1 (pbest_{pi} -y_{pi}) + c_2. r_2 (gbest_{pi} -y_{pi})\\ y_{pi}(t+1)= & {} y_{pi}(t) + v_{pi}(t+1) \end{aligned}$$

where *t* is the number of iterations, *p* is the particle number, and *i* denotes the component number. $y_{pi}$ and $v_{pi}$ are particles’ positions and velocities respectively, and *w* is the inertia weight, which controls the effect of previous velocities on the present velocity, $c_1$ and $c_2$ are two positive constants, named cognitive learning rate and social learning rate, and $r_1$ and $r_2$ are uniformly distributed random numbers between 0 and 1. The *p*th particle’s local best solution is $pbest_{pi}$, while $gbest_{pi}$ represents the global best. Once the best position of all particles are improved after a significant number of generations, the PSO algorithm terminates.

### Grey Wolf Optimization (GWO)

In 2014, Mirijalili et al.^60^ presented GWO, a population-based meta heuristic algorithm that simulates grey wolf leadership and hunting. The top predators in the food chain are grey wolves. It lives in packs of 5–12 members. The first social hierarchy level is alpha ($\alpha$). The alpha wolf makes judgements about hunting, employment, sleeping, and more. Pack members must follow the alpha wolf’s directions. Beta ($\beta$) is the second level. Alpha wolves make decisions with beta wolves’ aid. The beta supports the alpha’s orders and provides feedback. Delta ($\delta$) wolves are subordinates to the alpha and beta wolves but dominate the omega ($\omega$) wolves, the lowest level in the pack’s social order. Omega wolves are the pack’s scapegoat and must submit to all superior wolves. Alpha, beta, and delta are the first, second, and third fittest solutions in grey wolf optimization (GWO). Omega represents the remaining solutions. Grey wolves surround prey during hunting, this encircling behaviour is expressed mathematically in the following equation.

$$\begin{aligned} D= & {} |C.y_{P} (t) - A.y(t)|\\ y(t+1)= & {} y_{P} (t) - A.D \end{aligned}$$

where A and C are coefficient vectors, *t* is the current iteration, $y_P$ is the position vector of the prey, and *y* indicates the grey wolf’s position vector. A & C vectors are calculated as follows:

$$\begin{aligned} A= & {} 2a r_1 - a; a = 2~(1 - \frac{\text {Iteration}}{\text {Max Iteration}})\\ C= & {} 2 r_2 \end{aligned}$$

where $r_1$, $r_2$ are random vectors in [0,1] and $a's$ components drop linearly from 2 to 0. We assumed the alpha, beta, and delta have higher prey—locating knowledge in the grey wolf mathematical model. The first three best solutions are stored, and the other agents must update their positions based on the best search agent’s position as stated in the following equations.

$$\begin{aligned} D_\alpha= & {} |C_{1}.y_{\alpha }-y|; ~~D_\beta = |C_{2}.y_{\beta }-y|; ~~D_\delta = |C_{3}.y_{\delta }-y|\\ y_1= & {} y_{\alpha } - A_{1}.D_{\alpha }; ~~y_2 = y_{\beta } - A_{2} D_\beta ; ~~y_3 = y_{\delta } - A{_3} D_\delta \\ y(t+1)= & {} \frac{(y_1 + y_2 + y_3)}{3}. \end{aligned}$$

When prey stops moving, the grey wolf attacks. A is a random vector in [-2a, 2a]. When $|A| < 1$, the wolves exploit the prey, but when $|A| > 1$, they must diverge to find a better prey.

### Social Group Optimization (SGO)

SGO is a new optimization method that relies on humans’ group-solving abilities, introduced by Satapathy and Naik^61^ in 2016. In this approach, a group of people are selected and enhanced with knowledge of diverse capacities to solve a function. SGO contains two phases, the Improving and Acquiring phase that helps to find optimal solution for numerous real-life problems.

#### Improving phase

In this phase, the best person in the group raises everyone’s knowledge level and is represented as follows:

$$\begin{aligned} y_{new} = C.y_{old} + r(y_{best} - y_{old}) \end{aligned}$$

where C is the self-introspection parameter and $C\in [0~~1]$, r is the random number between 0 and 1, $y_{new}$, $y_{old}$ & $y_{best}$ are the group’s new, old, and best individual’s positions respectively.

#### Acquiring phase

In this phase, each group member learns from the best and other group members. The new solution is generated with the help of partner’s position $y_p$. The present study deals with the minimization problem, and the mathematical expression is described as follows:

$$\begin{aligned} y_{new}= & {} y_{old}+ r_1(y- y_p) + r_2( y_{best} -y) ~~if~~ Z < Z_p \\ y_{new}= & {} y_{old} - r_1(y- y_p) + r_2 ( y_{best} -y) ~~if~~ Z > Z_p \end{aligned}$$

where $r_1$, $r_2 \in [0~~1]$, which improves social behaviour by promoting the algorithm’s randomness. In this scenario, $y_{old}$ is the individuals’ improvement phase position. If $y_{new}$ improves objective function fitness, then it is accepted.

### Neutrosophic approach in MOOP

The complexity of real life often leads to undecidability when making better decisions. The degree of indeterminacy plays a significant role in the decision-making process, in addition to the acceptance and rejection degrees. As such, the feasible solution set includes the indeterminacy degree. NS is unique among uncertain decision sets since it has an independent degree of indeterminacy, which sets it apart from fuzzy and intuitionistic fuzzy sets. A neutrosophic decision set $(G_N)$ for the MOOP is formed by the intersection of integrated neutrosophic objects $(O_i)$ and neutrosophic constraints $(C_j)$. It is explained as follows:

$$\begin{aligned} G_N= & {} (\cap _{i=1}^{r} O_i ) (\cap _{j=1}^{m} C_j ) = (y,T_{G_N} (y),I_{G_N} (y),F_{G_N} (y))\\ T_{G_N}(y)= & {} \text {Min} \begin{Bmatrix} \mu _{O1}(y),&\mu _{O2}(y),&\dots ,&\mu _{Or}(y)\\ \mu _{C1}(y),&\mu _{C2}(y),&\dots ,&\mu _{Cm}(y) \end{Bmatrix} \\ I_{G_N}(y)= & {} \text {Max} \begin{Bmatrix} \sigma _{O1}(y),&\sigma _{O2}(y),&\dots ,&\sigma _{Or}(y)\\ \sigma _{C1}(y),&\sigma _{C2}(y),&\dots ,&\sigma _{Cm}(y) \end{Bmatrix} \\ F_{G_N}(y)= & {} \text {Max} \begin{Bmatrix} \gamma _{O1}(y),&\gamma _{O2}(y),&\dots ,&\gamma _{Or}(y)\\ \gamma _{C1}(y),&\gamma _{C2}(y),&\dots ,&\gamma _{Cm}(y) \end{Bmatrix}, \forall ~~ y\in Y \end{aligned}$$

where, $T_{G_N} (y), I_{G_N} (y), F_{G_N} (y)$ represent the membership functions of truth, indeterminacy and falsity of decision set $G_N$. By minimizing the underdeviation of truth and the overdeviation of falsity and indeterminacy, the following proposed NGP is utilized to produce an achievement function for the MOOP.

### Proposed neutrosophic goal programming technique (NGP)

In approaches to neutrosophic goal programming, each marginal evaluation is converted into a neutrosophic membership goals in accordance with their highest accomplishment levels. The truth membership function can be attained up to a maximum of unity. The highest degree of accomplishment for the indeterminacy membership function is half. The utmost achievement degree for the falsehood membership function is zero. To create a Neutrosophic membership goals, the lower and upper limit of the truth, indeterminacy, and falsity membership functions are created as follows:

Maximization

$$\begin{aligned} U_i^\mu= & {} U^{acc} (O_i(y)), L_i^\mu = L^{acc}(O_i(y))\\ U_i^\sigma= & {} U_i^\mu -\alpha _k(U_i^\mu -L_i^\mu ), L_i^\sigma = L_i^\mu \\ U_i^\gamma= & {} U_i^\mu -\beta _k(U_i^\mu -L_i^\mu ), L_i^\gamma = L_i^\mu \\ \end{aligned}$$

Minimization

$$\begin{aligned} U_i^\mu= & {} U^{acc} (O_i(y)), L_i^\mu = L^{acc}(O_i(y))\\ U_i^\sigma= & {} U_i^\mu , L_i^\sigma = L_i^\mu -\alpha _k(U_i^\mu -L_i^\mu )\\ U_i^\gamma= & {} U_i^\mu , L_i^\gamma = L_i^\mu -\beta _k(U_i^\mu -L_i^\mu )\\ \end{aligned}$$

where $U^{acc}$, $L^{acc}$ are the upper and lower acceptance of the truth membership function, *y* is the decision variable, $(U_i^\mu , U_i^\sigma , U_i^\gamma )$ & $(L_i^\mu , L_i^\sigma , L_i^\gamma )$ are the upper and lower limits of truth, indeterminacy and falsity membership functions and $\alpha _K$ & $\beta _K$ are the scaling factors, as well as $\alpha _K$, $\beta _K\in [0 ~~ 1]$.

In multi objective goal programming problem (MOGPP), each objective function’s marginal evaluation is represented by its corresponding membership function. Since a linear type membership function has a simpler form and more understandable implications, it is generally the most comprehensive and commonly used type. The linear-type membership function takes the constant marginal rate of satisfaction or dissatisfaction degrees towards an objective into account. However, a non-linear membership function may be used to reflect each target’s desire level. The non-linear membership functions’ adaptable nature makes it possible to calculate the marginal assessment of the degree of objective satisfaction. It also depends on the values of a few parameters, which are sufficient to carry out the decision-maker’s plan effectively. Therefore, this study represents a fresh attempt to investigate the MOGPP in a neutrosophic setting with both linear and non-linear membership functions. The present study examines Linear, Hyperbolic, and Exponential membership functions. The truth, indeterminacy, and falsity membership functions of these three types are ($\mu _i^L, \sigma _i^L,\gamma _i^L$), ($\mu _i^H, \sigma _i^H,\gamma _i^H$), and ($\mu _i^E, \sigma _i^E,\gamma _i^E$) respectively, and they are described in the following sub sections.

### Linear membership functions

Maximization

$$\begin{aligned} \mu _{i}^{L}(O_{i}(y))&= {\left\{ \begin{array}{ll} 1, &{} O_{i}(y)\ge U_{i}^{\mu } \\ \frac{O_{i}(y) - L_{i}^{\mu }}{U_{i}^{\mu } - L_{i}^{\mu }}, &{} L_{i}^{\mu }< O_{i}(y)<U_{i}^{\mu } \\ 0, &{} O_{i}(y)\le L_{i}^{\mu } \end{array}\right. } \\ \sigma _{i}^{L}(O_{i}(y))&= {\left\{ \begin{array}{ll} 0, &{} O_{i}(y)\ge U_{i}^{\sigma } \\ \frac{U_{i}^{\sigma } - O_{i}(y)}{U_{i}^{\sigma } - L_{i}^{\sigma }}, &{} L_{i}^{\sigma }< O_{i}(y)<U_{i}^{\sigma } \\ 1, &{} O_{i}(y)\le L_{i}^{\sigma } \end{array}\right. } \end{aligned}$$

1 $$\begin{aligned} \gamma _{i}^{L}(O_{i}(y))= {\left\{ \begin{array}{ll} 0, &{} O_{i}(y)\ge U_{i}^{\gamma } \\ \frac{U_{i}^{\gamma }-O_{i}(y)}{U_{i}^{\gamma }-L_{i}^{\gamma }}, &{} L_{i}^{\gamma }< O_{i}(y)<U_{i}^{\gamma } \\ 1, &{} O_{i}(y)\le L_{i}^{\gamma } \end{array}\right. } \end{aligned}$$

Minimization

$$\begin{aligned} \mu _{i}^{L}(O_{i}(y))&= {\left\{ \begin{array}{ll} 0, &{} O_{i}(y)\ge U_{i}^{\mu } \\ \frac{U_{i}^{\mu }-O_{i}(y)}{U_{i}^{\mu }-L_{i}^{\mu }}, &{} L_{i}^{\mu }< O_{i}(y)<U_{i}^{\mu } \\ 1, &{} O_{i}(y)\le L_{i}^{\gamma } \end{array}\right. } \\ \sigma _{i}^{L}(O_{i}(y))&= {\left\{ \begin{array}{ll} 1, &{} O_{i}(y)\ge U_{i}^{\sigma } \\ \frac{O_{i}(y)-L_{i}^{\sigma }}{U_{i}^{\sigma }-L_{i}^{\sigma }}, &{} L_{i}^{\sigma }< O_{i}(y)<U_{i}^{\sigma } \\ 0, &{} O_{i}(y)\le L_{i}^{\sigma } \end{array}\right. } \end{aligned}$$

2 $$\begin{aligned} \gamma _{i}^{L}(O_{i}(y))= {\left\{ \begin{array}{ll} 1, &{} O_{i}(y)\ge U_{i}^{\gamma } \\ \frac{O_{i}(y)-L_{i}^{\gamma }}{U_{i}^{\gamma }-L_{i}^{\gamma }}, &{} L_{i}^{\gamma }< O_{i}(y)<U_{i}^{\gamma } \\ 0, &{} O_{i}(y)\le L_{i}^{\gamma } \end{array}\right. } \end{aligned}$$

### Exponential membership functions

Maximization

$$\begin{aligned} \mu _{i}^{E}(O_{i}(y))&= {\left\{ \begin{array}{ll} 1, &{} O_{i}(y)\ge U_{i}^{\mu } \\ \frac{e^{-d}\biggl (\frac{U_{i}^{\mu }-O_{i}(y)}{U_{i}^{\mu }-L_{i}^{\mu }}\biggr ) - e^{-d}}{1 - e^{-d}}, &{} L_{i}^{\mu }< O_{i}(y)<U_{i}^{\mu } \\ 0, &{} O_{i}(y)\le L_{i}^{\mu } \end{array}\right. } \\ \sigma _{i}^{E}(O_{i}(y))&= {\left\{ \begin{array}{ll} 0, &{} O_{i}(y)\ge U_{i}^{\sigma } \\ \frac{e^{-d}\biggl (\frac{O_{i}(y)-L_{i}^{\sigma }}{U_{i}^{\sigma }-L_{i}^{\sigma }}\biggr ) - e^{-d}}{1-e^{-d}}, &{} L_{i}^{\sigma }< O_{i}(y)<U_{i}^{\sigma } \\ 1, &{} O_{i}(y)\le L_{i}^{\sigma } \end{array}\right. } \end{aligned}$$

3 $$\begin{aligned} \gamma _{i}^{E}(O_{i}(y))= {\left\{ \begin{array}{ll} 0, &{} O_{i}(y)\ge U_{i}^{\gamma } \\ \frac{e^{-d}\biggl (\frac{O_{i}(y)-L_{i}^{\gamma }}{U_{i}^{\gamma }-L_{i}^{\gamma }}\biggr ) - e^{-d}}{1-e^{-d}}, &{} L_{i}^{\gamma }< O_{i}(y)<U_{i}^{\gamma } \\ 1, &{} O_{i}(y)\le L_{i}^{\gamma } \end{array}\right. } \end{aligned}$$

Minimization

$$\begin{aligned} \mu _{i}^{E}(O_{i}(y))&= {\left\{ \begin{array}{ll} 0, &{} O_{i}(y)\ge U_{i}^{\mu } \\ \frac{e^{-d}\biggl (\frac{O_{i}(y)-L_{i}^{\mu }}{U_{i}^{\mu }-L_{i}^{\mu }}\biggr ) - e^{-d}}{1-e^{-d}}, &{} L_{i}^{\mu }< O_{i}(y)<U_{i}^{\mu } \\ 1, &{} O_{i}(y)\le L_{i}^{\mu } \end{array}\right. } \\ \sigma _{i}^{E}(O_{i}(y))&= {\left\{ \begin{array}{ll} 1, &{} O_{i}(y)\ge U_{i}^{\sigma } \\ \frac{e^{-d}\biggl (\frac{U_{i}^{\sigma }-O_{i}(y)}{U_{i}^{\sigma }-L_{i}^{\sigma }}\biggr ) - e^{-d}}{1-e^{-d}}, &{} L_{i}^{\sigma }< O_{i}(y)<U_{i}^{\sigma } \\ 0, &{} O_{i}(y)\le L_{i}^{\sigma } \end{array}\right. } \end{aligned}$$

4 $$\begin{aligned} \gamma _{i}^{E}(O_{i}(y))= {\left\{ \begin{array}{ll} 1, &{} O_{i}(y)\ge U_{i}^{\gamma } \\ \frac{e^{-d}\biggl (\frac{U_{i}^{\gamma }-O_{i}(y)}{U_{i}^{\gamma }-L_{i}^{\gamma }}\biggr ) - e^{-d}}{1-e^{-d}}, &{} L_{i}^{\gamma }< O_{i}(y)<U_{i}^{\gamma } \\ 0, &{} O_{i}(y)\le L_{i}^{\gamma } \end{array}\right. } \end{aligned}$$

### Hyperbolic membership function

Maximization

$$\begin{aligned} \mu _{i}^{H}(O_{i}(y))&= {\left\{ \begin{array}{ll} 1, &{} O_{i}(y)\ge U_{i}^{\mu } \\ \frac{1}{2}\biggl [1+\tanh {\biggl (\theta _i \biggl (O_{i}(y)-\frac{U_{i}^{\mu }+L_{i}^{\mu }}{2}}\biggr )\biggr )\biggr ], &{} L_{i}^{\mu }< O_{i}(y)<U_{i}^{\mu } \\ 0, &{} O_{i}(y)\le L_{i}^{\mu } \end{array}\right. } \\ \sigma _{i}^{H}(O_{i}(y))&= {\left\{ \begin{array}{ll} 0, &{} O_{i}(y)\ge U_{i}^{\sigma } \\ \frac{1}{2}\biggl [1+\tanh {\biggl (\theta _i \biggl (\frac{U_{i}^{\sigma }+L_{i}^{\sigma }}{2}-O_{i}(y)}\biggr )\biggr )\biggr ], &{} L_{i}^{\sigma }< O_{i}(y)<U_{i}^{\sigma } \\ 1, &{} O_{i}(y)\le L_{i}^{\sigma } \end{array}\right. } \end{aligned}$$

5 $$\begin{aligned} \gamma _{i}^{H}(O_{i}(y))= {\left\{ \begin{array}{ll} 0, &{} O_{i}(y)\ge U_{i}^{\gamma } \\ \frac{1}{2}\biggl [1+\tanh {\biggl (\theta _i \biggl (\frac{U_{i}^{\gamma }+L_{i}^{\gamma }}{2}-O_{i}(y)}\biggr )\biggr )\biggr ], &{} L_{i}^{\gamma }< O_{i}(y)<U_{i}^{\gamma } \\ 1, &{} O_{i}(y)\le L_{i}^{\gamma } \end{array}\right. } \end{aligned}$$

Minimization

$$\begin{aligned} \mu _{i}^{H}(O_{i}(y))&= {\left\{ \begin{array}{ll} 0, &{} O_{i}(y)\ge U_{i}^{\mu } \\ \frac{1}{2}\biggl [1+\tanh {\biggl (\theta _i \biggl (\frac{U_{i}^{\mu }+L_{i}^{\mu }}{2}-O_{i}(y)}\biggr )\biggr )\biggr ], &{} L_{i}^{\mu }< O_{i}(y)<U_{i}^{\mu } \\ 1, &{} O_{i}(y)\le L_{i}^{\mu } \end{array}\right. } \\ \sigma _{i}^{H}(O_{i}(y))&= {\left\{ \begin{array}{ll} 1, &{} O_{i}(y)\ge U_{i}^{\sigma } \\ \frac{1}{2}\biggl [1+\tanh {\biggl (\theta _i \biggl (O_{i}(y)-\frac{U_{i}^{\sigma }+L_{i}^{\sigma }}{2}}\biggr )\biggr )\biggr ], &{} L_{i}^{\sigma }< O_{i}(y)<U_{i}^{\sigma } \\ 0, &{} O_{i}(y)\le L_{i}^{\sigma } \end{array}\right. } \end{aligned}$$

6 $$\begin{aligned} \gamma _{i}^{H}(O_{i}(y))= {\left\{ \begin{array}{ll} 1, &{} O_{i}(y)\ge U_{i}^{\gamma } \\ \frac{1}{2}\biggl [1+\tanh {\biggl (\theta _i \biggl (O_{i}(y)-\frac{U_{i}^{\gamma }+L_{i}^{\gamma }}{2}}\biggr )\biggr )\biggr ], &{} L_{i}^{\gamma }< O_{i}(y)<U_{i}^{\gamma } \\ 0, &{} O_{i}(y)\le L_{i}^{\gamma } \end{array}\right. } \end{aligned}$$

where $\theta _i = \frac{6}{U_i-L_i}, i = 1, 2,\ldots , r$. For all objective functions, $U_i \ne L_i$. If $U_i= L_i$, in any circumstance then the membership value will be assumed to be 1.

In order to highest accomplishment level of truth, indeterminacy and falsity, the membership functions mentioned in Eqs. (1) to (6) are converted into the neutrosophic goal as follows:

$$\begin{aligned} \mu _i^T (O_{i}(y)) + d_{iT}^{-} - d_{iT}^{+}= & {} 1\\ \sigma _{i}^I (O_{i}(y)) + d_{iI}^{-} - d_{iI}^{+}= & {} 0.5\\ \gamma _i^F (O_{i}(y)) + d_{iF}^{-} - d_{iF}^{+}= & {} 0 \end{aligned}$$

where $( d_{iT}^-, d_{iT}^+)$; $(d_{iI}^-, d_{iI}^+)$ and $(d_{iF}^-, d_{iF}^+)$ are under and over deviational variables of truth, indeterminacy and falsity membership functions respectively, $(\mu _i^T \sigma _{i}^I, \gamma _i^F)$ is the truth, indeterminacy and falsity membership functions. Also $d_{iT}^-. d_{iT}^+ = 0$; $d_{iI}^-.d_{iI}^+ = 0$ & $d_{iF}^-. d_{iF}^+ = 0$. Other researchers produced the achievement function for the goal programming problem by minimizing the over deviation of the falsity membership functions and the under deviations of the truth and indeterminacy group. However, the decision-maker in this study minimized the over achievement of the indeterminacy and falsity membership functions and the under achievement of truth to generate the achievement function. As a result, the neutrosophic goal programming problem’s achievement function is expressed as follows:

$$\begin{aligned}{} & {} \text {Min}~ O = \sum _{i=1}^{r}w_{iT}d_{iT}^{-} + \sum _{i=1}^{r}w_{iI}d_{iI}^{+} + \sum _{i=1}^{r}w_{iF}d_{iF}^{+}\\{} & {} \text {Subject to}\\{} & {} \mu _{i}^T (O_{i}(y)) + d_{iT}^{-}\ge 1\\{} & {} \sigma _{i}^{I}(O_{i}(y)) - d_{iI}^{+} \le 0.5\\{} & {} \gamma _{i^F}(O_{i}(y)) - d_{iF}^{+} \le 0\\{} & {} \mu _i^T (O_{i}(y)) \ge \sigma _i^I (O_{i}(y))\\{} & {} \mu _i^T (O_{i}(y)) \ge \gamma _i^F (O_{i}(y))\\{} & {} \mu _i^T (O_{i}(y)) + \sigma _i^I(O_{i}(y)) + \gamma _i^F (O_{i}(y)) \le 3\\{} & {} d_{iT}^{-}. d_{iT}^{+} = 0; ~~d_{iI}^{-}. d_{iI}^{+} = 0 ~~ \& ~~ d_{iF}^{-}.d_{iF}^{+}=0.~~\forall ~~ i\\{} & {} h_{j}(y) \le \text {or} \ge \text {or} = b_{j}, j = 1, 2, \ldots , m \end{aligned}$$

7 $$\begin{aligned} y_i \ge 0, i = 1, 2,\ldots , n, \end{aligned}$$

where $w_{iT} = \frac{1}{(U_{i}^{\mu }-L_{i}^\mu )}$; $w_{iI} = \frac{1}{(U_{i}^{\sigma }-L_{i}^\sigma )}$; $w_{iF} =\frac{1}{(U_i^{\gamma }-L_i^{\gamma })}$ are the weights associated with the deviations of the membership goals. After the multi-objective optimization problem has been transformed into a single objective neutrosophic goal, bio-inspired algorithms GWO, PSO, and SGO are utilized to discover the best solution to the crisp neutrosophic goal programming problem. The crop land allocation problem mentioned in the study area is solved using the aforesaid methodology.

## Computational algorithm

It is possible to formulate a real-world scenario into an optimization model using a computational process for any mathematical model. Using the neutrosophic goal programming technique and a bio-inspired algorithm, the following steps are taken to solve the linear multi-objective crop land allocation problem.

Step 1: Consider the multi-objective linear programming problem (MLPP) with ‘r’ objectives, ‘m’ constraints, and ‘n’ decision variables as follows:

$$\begin{aligned}{} & {} \text {Max/Min} {O_1, O_2\dots , O_r}\\{} & {} \text {Subject to}\\{} & {} h_{j}(y) \le \text {or} \ge \text {or} = b_{j}, j = 1, 2, \ldots , m \\{} & {} y_i \ge 0, i = 1, 2,\ldots , n. \end{aligned}$$

Step 2: Utilizing the provided constraints, determine each objective function’s solution one at a time. Create a pay-off matrix using the optimum solution’s information as follows:

$$\begin{aligned} \begin{bmatrix} O_1^{*}(y^1) &{} O_{2}(y^1) &{} \cdots &{} O_{r}(y^1)\\ O_{2}(y^2) &{} O_2^{*}(y^2) &{} \cdots &{} O_{r}(y^2)\\ \vdots &{} \vdots &{} \ddots &{} \vdots \\ O_{1}(y^r)&{} O_{2}(y^r) &{} \cdots &{} O_r^{*}(y^r) \end{bmatrix} \end{aligned}$$

where $(y^1),(y^2),\ldots (y^r)$ are the solution sets of the objectives $O_1, O_2,\ldots O_r$ respectively.

Step 3: Obtain the lower and upper bound of each objective functions and determine the lower and upper bound of truth, indeterminacy & falsity membership function.

Step 4: Create Linear, Exponential and Hyperbolic membership function by using the bounds mentioned in step 3.

Step 5: As stated in Eq. (7), construct a neutrosophic achievement function and goals using the membership functions generated in Step 4.

Step 6: Use bio-inspired algorithms such as GWO, PSO, SGO to find the optimal solution of the goal mentioned in step 5.

## Results and discussion

In this study, the parameters of production, profit, expenditure, labour, and water requirements of the problem explained in the study area are regarded as hexagonal intuitionistic fuzzy numbers due to uncertainty and indeterminacy. The hexagonal intutionistic fuzzy numbers for expenditure, production, profit and labour per acre, water, and labour availability per season are shown from Table 1, 2, 3 and 4. The estimated value of the hexagonal intuitionistic fuzzy numbers are derived from the Thamaraiselvi approach^62^. Once the hexagonal intutionistic fuzzy numbers are converted into the estimated value, the objectives (production, profit, labour cost, seed cost, fertilizer and pesticides cost, and miscellaneous costs) and the constraints (land, water, labour, and food requirements) are modified as follows:

**Table 1:** Labour cost and seed costs’ hexagonal intuitionistic parameter.

Crop	Labour cost	Seed cost
Paddy $(y_1)$	(8100,8700,9000,9300,9600,10200;	(3000,3100,3150,3200,3250,3350;
	7800,8400,9000,9300,9900,10500)	2950,3050,3150,3200,3300,3400)
Ground nut $(y_2)$	(4500,5100,5300,5700,6000,6600;	(4325,4425,4475,4525,4575,4675;
	4200,4800,5300,5700,6300,6900)	4275,4375,4475,4525,4625,4725)
Cotton $(y_3)$	(18600,19200,19500,19800,20100,20700;	(1325,1425,1475,1525,1575,1675;
	18300,18900,19500,19800,20400,21000)	1275,1375,1475,1525,1625,1725)
Paddy$(y_4)$	(8100,8700,9000,9300,9600,10200;	(3000,3100,3150,3200,3250,3350;
	7800,8400,9000,9300,9900,10500)	2950,3050,3150,3200,3300,3400)
Ground nut$(y_5)$	(4500,5100,5300,5700,6000,6600;	(4325,4425,4475,4525,4575,4675;
	4200,4800,5300,5700,6300,6900)	4275,4375,4475,4525,4625,4725)
Pearl millet$(y_6)$	(3600,4200,4500,4800,5100,5700;	(315,335,345,355,365,385;
	3300,3900,4500,4800,5400,6000)	305,325,345,355,375,395)
Sweet Corn$(y_7)$	(5100,5700,6000,6300,6600,7200;	(1825,1925,1975,2025,2075,2175;
	4800,5400,6000,6300,6900,7500)	1775,1875,1975,2025,2125,2225)
Paddy$(y_8)$	(8100,8700,9000,9300,9600,10200;	(3000,3100,3150,3200,3250,3350;
	7800,8400,9000,9300,9900,10500)	2950,3050,3150,3200,3300,3400)
Brinjal$(y_9)$	(7800,8400,8700,9000,9300,9900;	(1425,1525,1575,1625,1675,1775;
	7500,8100,8700,9000,9600,10200)	1375,1475,1575,1625,1725,1825)
Onion$(y_{10})$	(9600,10200,10500,10800,11100,11700;	(5825,5925,5975,6025,6075,6175;
	9300,9900,10500,10800,11400,12000)	5775,5875,5975,6025,6125,6225)
Sesame$(y_{11})$	(3375,3675,3825,3975,4125,4425;	(615,635,645,655,665,685;
	3225,3525,3825,3975,4275,4575)	605,625,645,655,675,695)

**Table 2:** Pesticides and fertiliser cost and miscellaneous costs’ hexagonal intuitionistic parameter.

Crop	Pesticides & Fertilizer cost	Miscellaneous cost
Paddy $(y_1)$	(3135,3235,3285,3335,3385,3485;	(3565,3765,3865,3965,4065,4265;
	3085,3185,3285,3335,3435,3535)	3465,3665,3865,3965,4165,4365)
Ground nut $(y_2)$	(2325,2425,2475,2525,2575,2675;	(3650,3850,3950,4050,4150,4350;
	2275,2375,2475,2525,2625,2725)	3550,3750,3950,4050,4250,4450)
Cotton $(y_3)$	(4825,4925,4975,5025,5075,5175;	(3050,3250,3350,3450,3550,3750;
	4775,4875,4975,5025,5125,5225)	2950,3150,3350,3450,3650,3850)
Paddy $(y_4)$	(3135,3235,3285,3335,3385,3485;	(3565,3765,3865,3965,4065,4265;
	3085,3185,3285,3335,3435,3535)	3465,3665,3865,3965,4165,4365)
Ground nut $(y_5)$	(2325,2425,2475,2525,2575,2675;	(3650,3850,3950,4050,4150,4350;
	2275,2375,2475,2525,2625,2725)	3550,3750,3950,4050,4250,4450)
Pearl millet $(y_6)$	(1005,1105,1155,1205,1255,1355;	(800,1000,1100,1200,1300,1500;
	955,1055,1155,1205,1305,1405)	700,900,1100,1200,1400,1600)
Sweet Corn $(y_7)$	(2625,2725,2775,2825,2875,2975;	(750,950,1050,1150,1250,1450;
	2575,2675,2775,2825,2925,3025)	650,850,1050,1150,1350,1550)
Paddy $(y_8)$	(3135,3235,3285,3335,3385,3485;	(3565,3765,3865,3965,4065,4265;
	3085,3185,3285,3335,3435,3535)	3465,3665,3865,3965,4165,4365)
Brinjal $(y_9)$	(5325,5425,5475,5525,5575,5675;	(3250,3450,3550,3650,3750,3950;
	5275,5375,5475,5525,5625,5725)	3150,3350,3550,3650,3850,4050)
Onion $(y_{10})$	(4825,4925,4975,5025,5075,5175;	(1550,1750,1850,1950,2050,2250;
	4775,4875,4975,5025,5125,5225)	1450,1650,1850,1950,2150,2350)
Sesame $(y_{11})$	(1205,1305,1355,1405,1455,1555;	(2270,2470,2570,2670,2770,2970;
	1155,1255,1355,1405,1505,1605)	2170,2370,2570,2670,2870,3070)

**Table 3:** Hexagonal intuitionistic fuzzy number for water and labour availability.

Season	Water availability	Labour availability
Kharif	(21800,24100,25250,26400,27550,29850;	(256,258,259,260,261,263;
	20650,22950,25250,26400,28700,31000)	255,257,259,260,262,264)
Rabi	(20500,23000,24250,25500,26750,29250;	(256,258,259,260,261,263;
	19250,21750,24250,25500,28000,30500)	255,257,259,260,262,264)
Summer	(20500,23000,24250,25500,26750,29250;	(256,258,259,260,261,263;
	19250,21750,24250,25500,28000,30500)	255,257,259,260,262,264)

$$\text {Max} O_1=3430y_1+1555y_2+1275y_3+3760y_4+1455y_5+1365y_6+2730y_7+2830y_8+11600y_9+9650y_{10}+355y_{11}$$

$$\text {Max} O_2=73745y_1+98742.5y_2+74268.75y_3+80840y_4+92392.5y_5+29347.5y_6+50505y_7+60845y_8+232000y_9+154400y_{10}+24335.25y_{11}$$

$$\text {Min} O_3 = 9150y_1+5550y_2+19650y_3+9150y_4+5550y_5+4650y_6+6150y_7+ 9150y_8+8850y_9+10650y_{10}+3900y_{11}$$

$$\text {Min} O_4=3175y_1+4500y_2+1500y_3+3175y_4+4500y_5+350y_6+2000y_7+ 3175y_8+1600y_9+6000y_{10}+650y_{11}$$

$$\text {Min} O_5=3310y_1+2500y_2+5000y_3+3310y_4+2500y_5+1180y_6+2800y_7+ 3310y_8+5500y_9+5000y_{10}+1380y_{11}$$

$$\text {Min} O_6=3915y_1+4000y_2+3400y_3+3915y_4+4000y_5+1150y_6+1100y_7+ 3915y_8+3600y_9+1900y_{10}+2620y_{11}$$

$$\begin{aligned}{} & {} \text {Subject to} \\{} & {} y_1+y_2+y_3\le 15 \\{} & {} y_3+y_4+y_5+y_6+y_7\le 15\\{} & {} y_8+y_9+y_{10}+y_{11}\le 15\\{} & {} y_1+y_2+y_3\le 25825\\{} & {} y_3+y_4+y_5+y_6+y_7\le 24875\\{} & {} y_8+y_9+y_{10}+y_{11}\le 24875\\{} & {} 30.5y_1+18.5y_2+65.5y_3\le 260\\{} & {} 30.5y_4+18.5y_5+15.5y_6+20.5y_7\le 260\\{} & {} 30.5y_8+29.5y_9+35.5y_{10}+13y_{11}\le 260\\{} & {} 3430y_1+3760y_4+2830y_8\ge 3760\\{} & {} 1365y_6\ge 230 \end{aligned}$$

8 $$\begin{aligned} y_1, y_2, \dots , y_{11}\ge 0 \end{aligned}$$

The individual maximum and minimum values of each objective function are taken from the pay-off matrix, which was derived by using step 2 of the computational algorithm, and they are (165983.2, 4651386, 234000, 167223.3, 118108.2, 156171.5) and (3990, 85785, 9933.516, 3233.974, 3508.829, and 4108.773), respectively. Also, the positive and negative ideal solutions of the objective functions are (165983.2, 4651386, 9933.516, 3233.974, 3508.829, 4108.773) and (3990, 85785, 234000, 167223.3, 118108.2, 156171.5) respectively. These positive and negative ideal solutions are used to form the membership functions for the present study. Following the generation of linear and non-linear membership functions, the NGP achievement function is produced.

By using a neutrosophic goal program mentioned in Eq. (7), with linear membership functions mentioned in Eqs. (1) and (2) the above Eq. (8) is converted into the following Eq. (9)

$$\begin{aligned}{} & {} \text {Min}~ O = \sum _{i=1}^{r}w_{iT}d_{iT}^{-} + \sum _{i=1}^{r}w_{iI}d_{iI}^{+} + \sum _{i=1}^{r}w_{iF}d_{iF}^{+}\\{} & {} \text {Subject to}\\{} & {} \frac{O_{i}(y) - L_{i}^{\mu }}{U_{i}^{\mu } - L_{i}^{\mu }} + d_{iT}^{-}\ge 1,~~ \text {for} ~~ i = 1, 2.\\{} & {} \frac{U_{i}^{\mu }-O_{i}(y)}{U_{i}^{\mu }-L_{i}^{\mu }} + d_{iT}^{-}\ge 1,~~ \text {for} ~~ i = 3, 4, 5, 6.\\{} & {} \frac{U_{i}^{\sigma } - O_{i}(y)}{U_{i}^{\sigma } - L_{i}^{\sigma }} - d_{iI}^{+} \le 0.5~~ \text {for} ~~ i = 1, 2.\\{} & {} \frac{O_{i}(y)-L_{i}^{\sigma }}{U_{i}^{\sigma }-L_{i}^{\sigma }} - d_{iI}^{+} \le 0.5~~ \text {for} ~~ i = 3, 4, 5, 6.\\{} & {} \frac{U_{i}^{\gamma }-O_{i}(y)}{U_{i}^{\gamma }-L_{i}^{\gamma }} - d_{iF}^{+} \le 0 ~~ \text {for} ~~ i = 1, 2.\\{} & {} \frac{O_{i}(y)-L_{i}^{\gamma }}{U_{i}^{\gamma }-L_{i}^{\gamma }} - d_{iF}^{+} \le 0 ~~ \text {for} ~~ 3, 4, 5, 6.\\{} & {} \mu _i^T (O_{i}(y)) \ge \sigma _i^I (O_{i}(y))\\{} & {} \mu _i^T (O_{i}(y)) \ge \gamma _i^F (O_{i}(y))\\{} & {} \mu _i^T (O_{i}(y)) + \sigma _i^I(O_{i}(y)) + \gamma _i^F (O_{i}(y)) \le 3\\{} & {} d_{iT}^{-}. d_{iT}^{+} = 0;~~ d_{iI}^{-}. d_{iI}^{+} = 0 ~~ \& ~~ d_{iF}^{-}.d_{iF}^{+}=0.~~\forall ~~ i \end{aligned}$$

9 $$\begin{aligned} \text {All the constraints of Eq.} (8). \end{aligned}$$

**Table 4:** Production, profit, labour’s hexagonal intuitionistic parameter.

Crop	Production	Profit	Labour
$y_1$	(3220,3340,3400,3460,3520,3640;	(69230,71810,73100,74390,75680,78260;	(27,29,30,31,32,34;
	3160,3280,3400,3460,3580,3700)	67940,70520,73100,74390,76970,79550)	26,28,30,31,33,35)
$y_2$	(1520,1540,1550,1560,1570,1590;	(96520,97790,98425,99060,99695,100965;	(15,17,18,19,20,22;
	1510,1530,1550,1560,1580,1600)	95885,97155,98425,99060,100330,101600)	14,16,18,19,21,23)
$y_3$	(1100,1200,1250,1300,1350,1450;	(64075,69900,72812.5,75725,78637.5,84462.5;	(62,64,65,66,67,69;
	1050,1150,1250,1300,1400,1500)	61162.5,66987.5,72812.5,75725,81550,87375)	61,63,65,66,68,70)
$y_4$	(3340,3580,3700,3820,3940,4180;	(71810,76970,79550,82130,84710,89870;	(27,29,30,31,32,34;
	3220,3460,3700,3820,4060,4300)	69230,74390,79550,82130,87290,92450)	26,28,30,31,33,35)
$y_5$	(1420,1440,1450,1460,1470,1490;	(90170,91440,92075,92710,93345,94615;	(15,17,18,19,20,22;
	1410,1430,1450,1460,1480,1500)	89535,90805,92075,92710,93980,95250)	14,16,18,19,21,23)
$y_6$	(1260,1320,1350,1380,1410,1470;	(27090,28380,29025,29670,30315,31605;	(12,14,15,16,17,19;
	1230,1290,1350,1380,1440,1500)	26445,27735,29025,29670,30960,32250)	11,13,15,16,18,20)
$y_7$	2520,2640,2700,2760,2820,2940;	(46620,48840,49950,51060,52170,54390;	(17,19,20,21,22,24;
	2460,2580,2700,2760,2880,3000)	45510,47730,49950,51060,53280,55500)	16,18,20,21,23,25)
$y_8$	(2620,2740,2800,2860,2920,3040;	(56330,58910,60200,61490,62780,65360;	(27,29,30,31,32,34;
	2560,2680,2800,2860,2980,3100)	55040,57620,60200,61490,64070,66650)	26,28,30,31,33,35)
$y_9$	(10900,11300,11500,11700,11900,12300;	(218000,226000,230000,234000,238000,246000;	(26,28,29,30,31,33;
	10700,11100,11500,11700,12100,12500)	214000,222000,230000,234000,242000,250000)	25,27,29,30,32,34)
$y_{10}$	(8600,9200,9500,9800,10100,10700;	(137600,147200,152000,156800,161600,171200;	(32,34,35,36,37,39;
	8300,8900,9500,9800,10400,11000)	132800,142400,152000,156800,166400,176000)	31,33,35,36,38,40)
$y_{11}$	(320,340,350,360,370,390;	(21936,23307,23992.5,24678,25363.5,26734.5;	11,12,12.5,13,13.5,14.5;
	310,330,350,360,380,400)	21250.5,22621.5,23992.5,24678,26049,27420)	10.5,11.5,12.5,13,14,15)

Using the neutrosophic goal program mentioned in Eq. (7), with exponential membership functions mentioned in Eqs. (3) and (4), Eq.(8) is converted into the following Eq. (10)

$$\begin{aligned}{} & {} \text {Min}~ O = \sum _{i=1}^{r}w_{iT}d_{iT}^{-} + \sum _{i=1}^{r}w_{iI}d_{iI}^{+} + \sum _{i=1}^{r}w_{iF}d_{iF}^{+}\\{} & {} \text {Subject to}\\ \end{aligned}$$

**Table 5:** Solutions of proposed method.

Crop	PSO			GWO			SGO		
	Linear	Exp	Hyper	Linear	Exp	Hyper	Linear	Exp	Hyper
$y_{1}$	0	0	1.1176	0	1.1666	0	0.2968	1.4136	0.7453
$y_{2}$	14.0541	14.0541	12.2115	13.988	12.1267	12.2699	13.5634	11.7020	12.8229
$y_{3}$	0	0	0	0	0	0	0	0	0
$y_{4}$	1	1	0	1.1062	0.1095	1	2.2304	0	1.6446
$y_{5}$	12.2642	12.2642	13.9116	12.0108	12.9788	13.0228	9.9528	12.5923	10.2618
$y_{6}$	0.1685	0.1685	0.17	0.1966	0.9493	0.3038	0.5045	0.3	1.2488
$y_{7}$	0	0	0	0	0	0	0	1.0915	0
$y_{8}$	0	0	0	0	0	0	0	0.5517	0
$y_{9}$	8.8136	8.8136	8.8132	8.7493	8.7771	8.6962	8.3099	8.5851	8.4844
$y_{10}$	0	0	0	0	0	0.1975	0	0	0
$y_{11}$	0	0	0	0	0	0	1.1367	0	0.6929
Max $O_{1}$	145926.3	145926.3	145528.8	145146.6	145493.5	144984.4	142463.8	144772.8	143980.2
Max $O_{2}$	4651397	4651397	4623190	4615950	4559832	4552538	4431406	4488069	4424082
Min $O_{3}$	234000.5	234000.5	233996.6	232760.6	233619.4	230001.9	233960.5	233241.7	233583.8
Min $O_{4}$	135768.1	135768.1	135263	134574.5	131569.8	132197.4	128058	130318.3	125931.6
Min $O_{5}$	117779.4	117779.4	117680.2	117011.7	116616.8	115716.8	115023.9	116545.1	114716.3
Min $O_{6}$	141110.9	141110.9	140790.8	140049.5	138199.5	137116.7	137432.8	135757.4	135490.6

**Table 6:** Net profit of farmer, proposed and existing approach.

Methods	Membership function	Net profi
	Linear	**4022723**
NPSO	Exponential	**4022723**
	Hyperbolic	3995460
	Linear	**3991554**
NGWO	Exponential	3939826
	Hyperbolic	3937505
	Linear	3816930
NSGO	Exponential	**3872206**
	Hyperbolic	3814360
FOT	Linear	3356462
IFOT	Linear	3356462
Torabi approach	Linear	3356462
Farmer’s output		2877399

$$\begin{aligned}{} & {} \frac{e^{-d}\biggl (\frac{U_{i}^{\mu } - O_{i}(y)}{U_{i}^{\mu } -L_{i}^{\mu }}\biggr ) - e^{-d}}{1-e^{-d}} + d_{iT}^{-}\ge 1,~~ \text {for} ~~ i = 1, 2.\\{} & {} \frac{e^{-d}\biggl (\frac{O_{i}(y)-L_{i}^{\mu }}{U_{i}^{\mu }-L_{i}^{\mu }}\biggr ) - e^{-d}}{1-e^{-d}} + d_{iT}^{-}\ge 1,~~ \text {for} ~~ i = 3, 4, 5, 6.\\{} & {} \frac{e^{-d}\biggl (\frac{O_{i}(y)-L_{i}^{\sigma }}{U_{i}^{\sigma }-L_{i}^{\sigma }}\biggr ) - e^{-d}}{1-e^{-d}} - d_{iI}^{+} \le 0.5~~ \text {for} ~~ i = 1, 2.\\{} & {} \frac{e^{-d}\biggl (\frac{U_{i}^{\sigma }-O_{i}(y)}{U_{i}^{\sigma }-L_{i}^{\sigma }}\biggr ) - e^{-d}}{1-e^{-d}} - d_{iI}^{+} \le 0.5~~ \text {for} ~~ i = 3, 4, 5, 6.\\{} & {} \frac{e^{-d}\biggl (\frac{O_{i}(y)-L_{i}^{\gamma }}{U_{i}^{\gamma }-L_{i}^{\gamma }}\biggr )- e^{-d}}{1-e^{-d}} -d_{iF}^{+} \le 0 ~~ \text {for} ~~ i = 1, 2.\\{} & {} \frac{e^{-d}\biggl (\frac{U_{i}^{\gamma }-O_{i}(y)}{U_{i}^{\gamma }-L_{i}^{\gamma }}\biggr ) - e^{-d}}{1-e^{-d}} - d_{iF}^{+} \le 0 ~~ \text {for} ~~ 3, 4, 5, 6.\\ \end{aligned}$$

**Figure 3:**
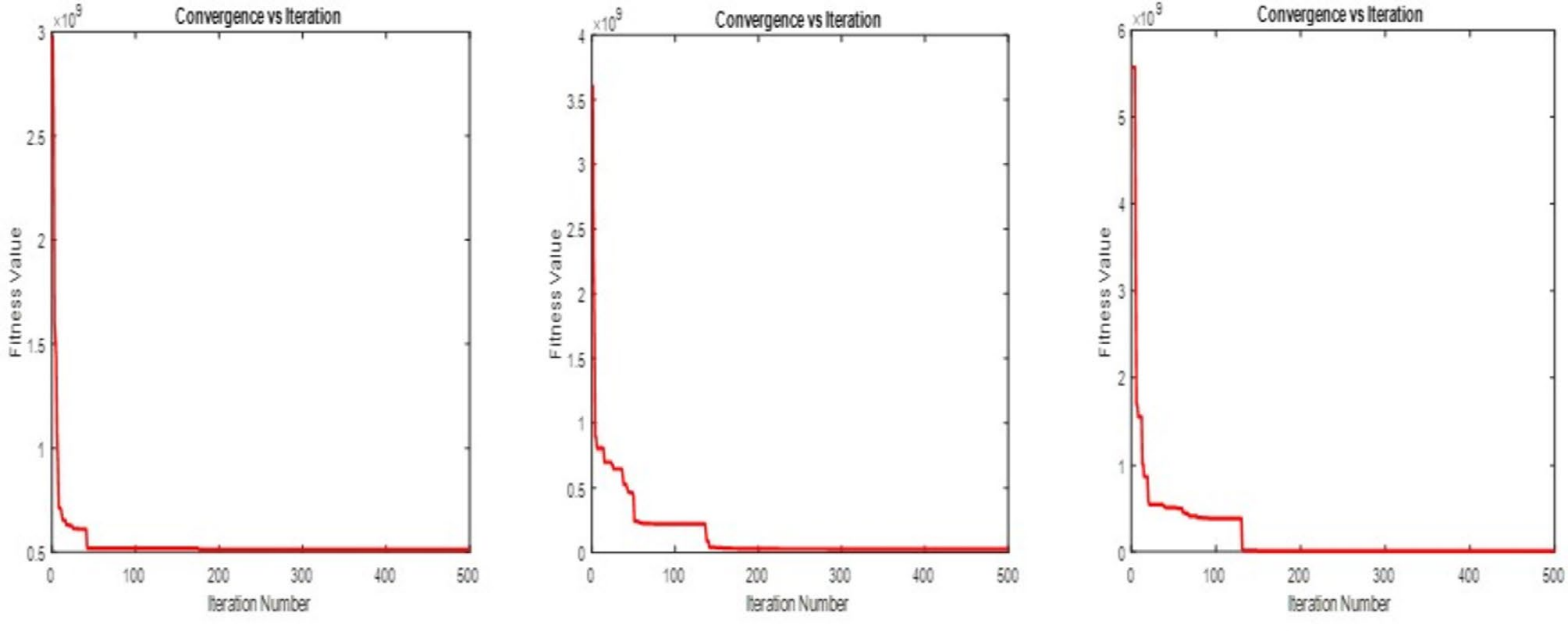
PSO Convergence of fitness value using linear, exponential and hyperbolic membership function.

**Figure 4:**
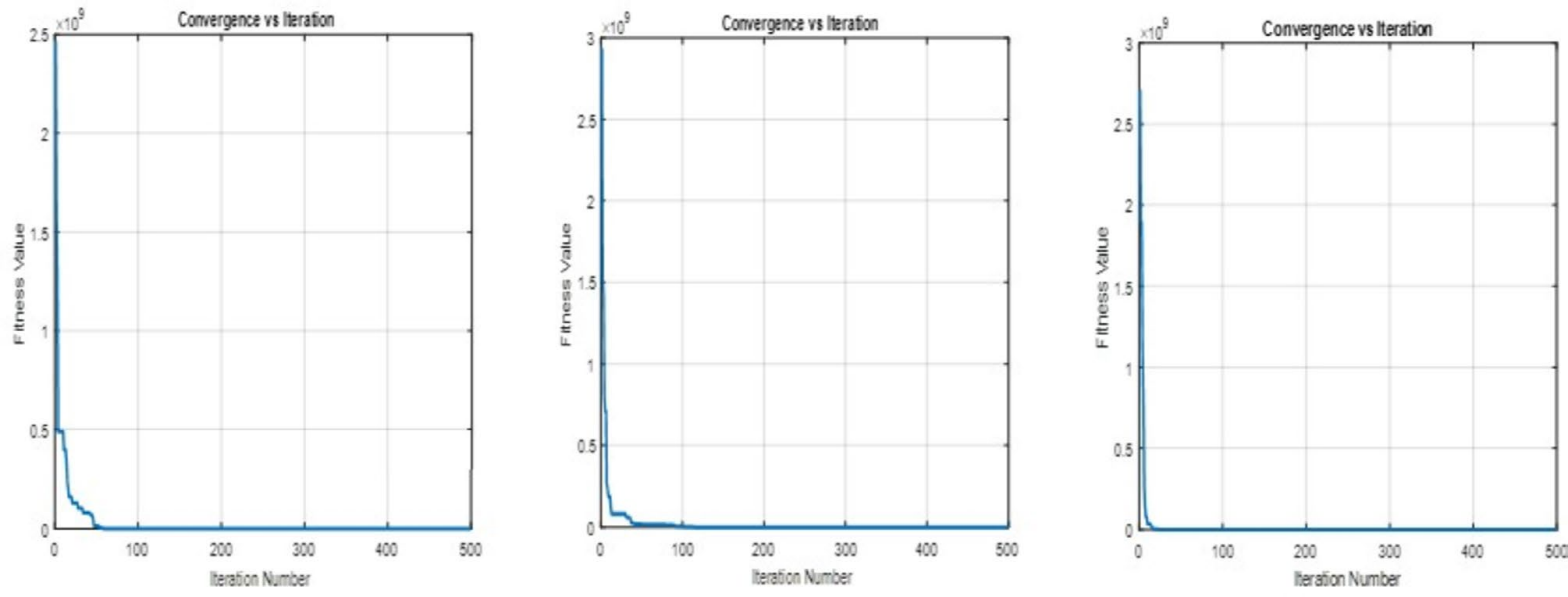
GWO Convergence of fitness value using linear, exponential and hyperbolic membership function.

**Figure 5:**
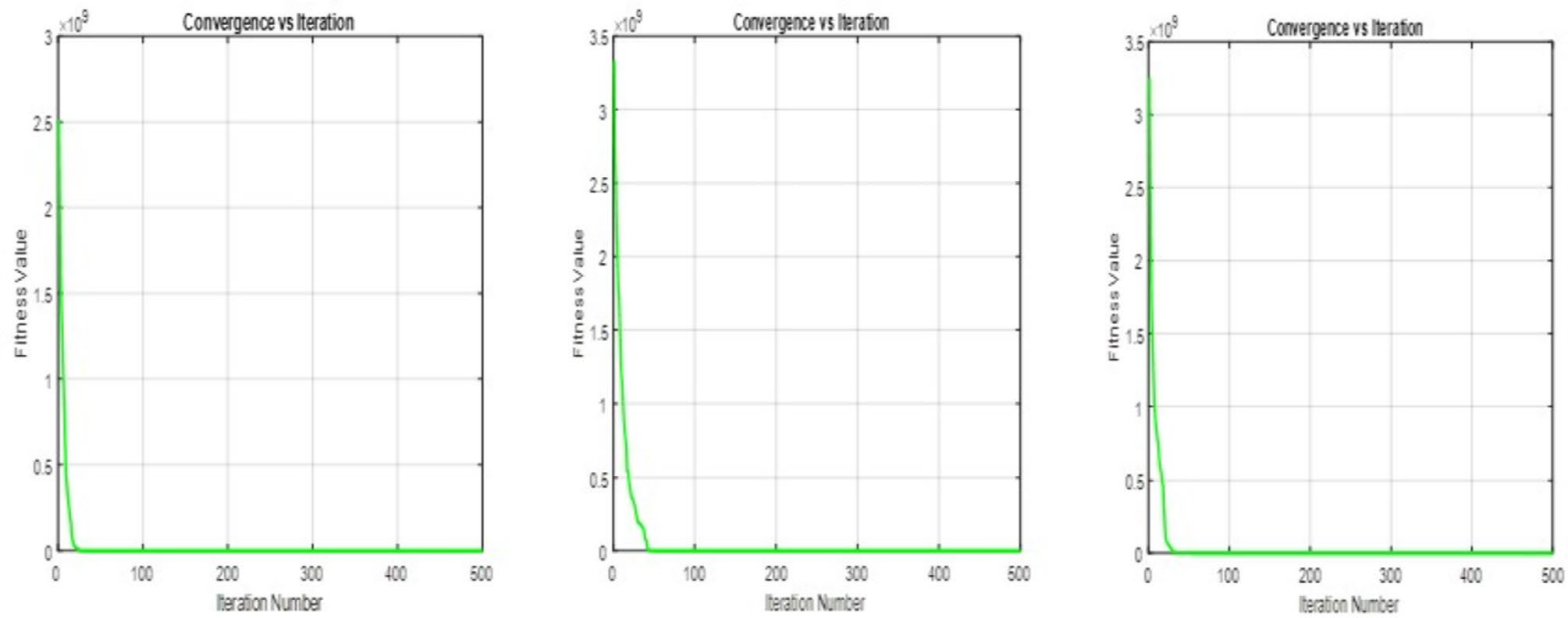
SGO Convergence of fitness value using linear, exponential and hyperbolic membership function.

$$\begin{aligned}{} & {} \mu _i^T (O_{i}(y)) \ge \sigma _i^I (O_{i}(y))\\{} & {} \mu _i^T (O_{i}(y)) \ge \gamma _i^F (O_{i}(y))\\{} & {} \mu _i^T (O_{i}(y)) + \sigma _i^I(O_{i}(y)) + \gamma _i^F (O_{i}(y)) \le 3\\{} & {} d_{iT}^{-}. d_{iT}^{+} = 0;~~ d_{iI}^{-}. d_{iI}^{+} = 0 ~~ \& ~~ d_{iF}^{-}.d_{iF}^{+}=0.~~\forall ~~ i \end{aligned}$$

10 $$\begin{aligned} \text {All the constraints of Eq.} (8). \end{aligned}$$

Using the neutrosophic goal program mentioned in Eq. (7), hyperbolic membership functions mentioned in Eqs. (5) and (6), Eq. (8) is converted into the following Eq. (11)

$$\begin{aligned}{} & {} \text {Min}~ O = \sum _{i=1}^{r}w_{iT}d_{iT}^{-} + \sum _{i=1}^{r}w_{iI}d_{iI}^{+} + \sum _{i=1}^{r}w_{iF}d_{iF}^{+}\\{} & {} \text {Subject to}\\{} & {} \frac{1}{2}\biggl [1+\tanh {\biggl (\theta _i \biggl (O_{i}(y)-\frac{U_{i}^{\mu }+L_{i}^{\mu }}{2}}\biggr )\biggr )\biggr ] + d_{iT}^{-}\ge 1,~~ \text {for} ~~ i = 1, 2.\\{} & {} \frac{1}{2}\biggl [1+\tanh {\biggl (\theta _i \biggl (\frac{U_{i}^{\mu }+L_{i}^{\mu }}{2}-O_{i}(y)}\biggr )\biggr )\biggr ] + d_{iT}^{-}\ge 1,~~ \text {for} ~~ i = 3, 4, 5, 6.\\{} & {} \frac{1}{2}\biggl [1+\tanh {\biggl (\theta _i \biggl (\frac{U_{i}^{\sigma }+L_{i}^{\sigma }}{2}-O_{i}(y)}\biggr )\biggr )\biggr ] - d_{iI}^{+} \le 0.5~~ \text {for} ~~ i = 1, 2.\\{} & {} \frac{1}{2}\biggl [1+\tanh {\biggl (\theta _i \biggl (O_{i}(y)-\frac{U_{i}^{\sigma }+L_{i}^{\sigma }}{2}}\biggr )\biggr )\biggr ] - d_{iI}^{+} \le 0.5~~ \text {for} ~~ i = 3, 4, 5, 6.\\{} & {} \frac{1}{2}\biggl [1+\tanh {\biggl (\theta _i \biggl (\frac{U_{i}^{\gamma }+L_{i}^{\gamma }}{2}-O_{i}(y)}\biggr )\biggr )\biggr ] - d_{iF}^{+} \le 0 ~~ \text {for} ~~ i = 1, 2.\\{} & {} \frac{1}{2}\biggl [1+\tanh {\biggl (\theta _i \biggl (O_{i}(y)-\frac{U_{i}^{\gamma }+L_{i}^{\gamma }}{2}}\biggr )\biggr )\biggr ] - d_{iF}^{+} \le 0 ~~ \text {for} ~~ 3, 4, 5, 6.\\{} & {} \mu _i^T (O_{i}(y)) \ge \sigma _i^I (O_{i}(y))\\{} & {} \mu _i^T (O_{i}(y)) \ge \gamma _i^F (O_{i}(y))\\{} & {} \mu _i^T (O_{i}(y)) + \sigma _i^I(O_{i}(y)) + \gamma _i^F (O_{i}(y)) \le 3\\{} & {} d_{iT}^{-}. d_{iT}^{+} = 0;~~ d_{iI}^{-}. d_{iI}^{+} = 0 ~~ \& ~~ d_{iF}^{-}.d_{iF}^{+}=0.~~\forall ~~ i \end{aligned}$$

11 $$\begin{aligned} \text {All the constraints of Eq.} (8). \end{aligned}$$

Using linear and non-linear membership functions as mentioned in Eqs. (1) to (6), the multi-objective optimization problem, which involves uncertainty and indeterminacy, is converted into the crisp single-objective neutrosophic optimization problem as mentioned in Eqs. (9) to (11). The objective functions of these three equations are called the achievement function of the problem. The main goal of this problem is to obtain the global optimal solution for these achievement functions. As mentioned earlier, bioinspired algorithms effectively search the search space and obtain the global optimal solution. Therefore, some of the bioinspired algorithms, namely PSO, GWO, and SGO, as explained under the methodology sections, are used to find the global optimal solution for the current problem. In this study, the number of populations, iterations, and runs for PSO, GWO, and SGO are fixed as the same, and they are (1000, 500, 20). Also, the acceleration constant (C1, C2) and the inertia weight w are fixed as (2, 2) and 1 in PSO, and the self-introspection parameter C is fixed as 0.25 in SGO. The optimal solutions of the achievement functions are obtained using these algorithms, and they converge to zero as the solution approaches 500 iterations in 20 runs. The convergence graphs for the three cases are shown in Figs. 3, 4 and 5. From these convergence graphs, it is concluded that PSO, GWO, and SGO provide the best compromise solution to the achievement functions. Therefore, it will provide the most satisfactory solution for the objective functions of production, profit, and expenditure. Also, the computation time of these three algorithms used to obtain the global optimal solution for the achievement functions of NGPs’ was almost the same. Thus, the best optimal solutions are classified by using the comparative analysis of the objective values, which is explained in the following section. For comparison, the optimal solutions of the objective functions are tabulated in Table 5. and the net profit obtained by the proposed and existing approaches and the farmer’s original net profit are tabulated in Table 6.

### Comparative analysis

**Figure 6:**
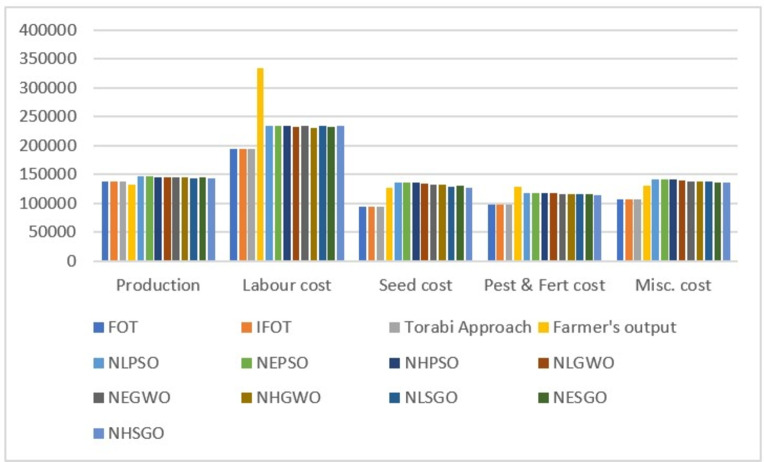
Comparative analysis of production and expenditure.

**Figure 7:**
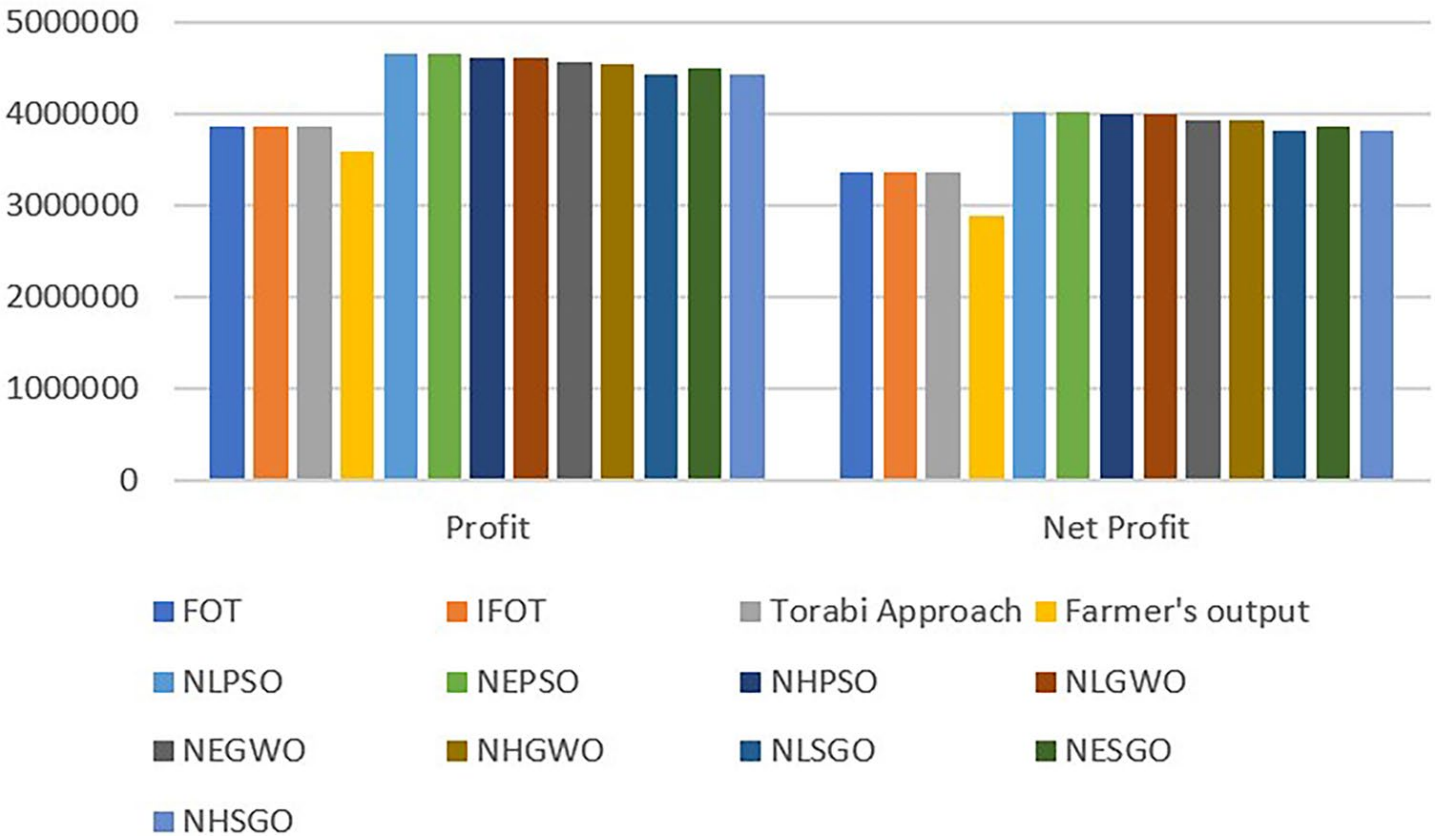
Comparative analysis of profit and net profit.

In this analysis, the performances of PSO, GWO, and SGO have been compared for the crop planning problem with the same population size and iterations (1000 and 500, respectively). The optimal (production, profit, labour cost, seed cost, fertilizer and pesticides, miscellaneous cost, and net profit) obtained by using PSO for the NGP, which was created by using linear, exponential, and hyperbolic membership functions, are (145926.3, 4651397, 234000.5, 135768.1, 117779.4, 141110.9, 4022723), (145926.3, 4651397, 234000.5, 135768.1, 117779.4, 141110.9, 4022723), and (145528.8, 4623190, 233996.6, 135263, 117680.2, 140790.8, 3995460), respectively. Compared to the net profit, PSO has provided a uniform and highly satisfactory solution to the NGP developed using linear and exponential membership functions. This is better than the net profit of the NGP generated using the hyperbolic membership function. Similarly, the optimal objective function values and net profit obtained using GWO and SGO for NGP generated using linear, exponential and hyperbolic membership functions are {(145146.6, 4615950, 232760.6, 134574.5, 117011.7, 140049.5, 3991554), (145493.5, 4559832, 233619.4, 131569.8, 116616.8, 138199.5, 3939826), (144984.4, 4552538, 230001.9, 132197.4, 115716.8, 137116.7, 3937505)} and {( 142463.8, 4431406, 233960.5, 128058, 115023.9, 137432.8, 3816930),( 144772.8, 4488069, 233241.7, 130318.3, 116545.1, 135757.4, 3872206),( 143980.2, 4424082, 233583.8, 125931.6, 114716.3, 135490.6, 3814360)} respectively. Comparing the net profit of NGP found using GWO, the net profit of NGP modeled with linear membership functions is higher than the net profit of NGP modeled with exponential and hyperbolic membership functions. Similarly, comparing the net profit of NGP found using SGO, the net profit of NGP modeled with exponential membership functions is higher than the net profit of NGP modeled with linear and hyperbolic membership functions. In the overall comparison, it is concluded that PSO provided a better net profit than GWO and SGO to the NGP. Also, for validation and superiority of the proposed method, its’ findings are compared with those of Zimmermann^63^, Angelov^64^, and Torabi^65^. The optimal net profit obtained from these three approaches is the same and is Rs. 3356462, and it is also compared with the farmer’s original net profit of Rs. 2877399. These comparisons demonstrate that the profit obtained by the proposed method is more abundant and acceptable than the profit obtained by the existing approaches and the actual profit of the farmers. The comparative analysis of four types of expenditure and production and the comparative analysis of profit and net profit are shown in Figs. 6 and 7.

## Conclusion

Numerous researchers have placed a great deal of emphasis on the crop planning problem due to its numerous risks and uncertainties. Without accounting for the indeterminacy component, a number of approaches have been devised to solve the land allocation problem. But in the current study, the achievement functions, which are generated by using a neutrosophic goal with independent linear, hyperbolic, and exponential truth, falsity, and indeterminacy membership functions, were solved in order to ascertain the optimal land allocation for the medium-sized farm-holders in the Ariyalur district. Additionally, PSO, GWO, and SGO were used to solve these achievement functions, and the optimal outputs are shown in Tables 5 and 6. Also, the comparative analysis of production and expenditure, profit, and net profit is shown in Figs. 6 and 7. From these tables and graphs, it is clear that the PSO algorithm provided a better compromise solution than the GWO and SGO for the NGP with a linear, hyperbolic, and exponential membership function. Also, the Zimmermann, Angelov, and Torabi and Hassini techniques were used to demonstrate the superiority of the proposed neutrosophic goal programming strategy. Comparing the results, the proposed strategy outperforms existing methods and the farmer’s original output. The proposed method optimizes medium-sized farm-holder land allocation. However, this technology can also be used to improve state and national irrigation systems, solve crop combination problems, and find the most effective and satisfying solutions to supply chain management, business, finance, and transportation problems utilising various bio-inspired algorithms. The proposed technique assumes expenditure, profit, and production as its objectives. Future research will likely include other goals for the benefit of farmers, such as reducing water use, maximizing organic fertilizer, and minimizing inorganic fertilizer. In this study, only PSO, SGO, and GWO have been used to optimize expenditure, profit, and production. However, a variety of bio-inspired algorithms are employed to determine the best solution for many MOOPs. Creating a generic neutrosophic framework with various bio-inspired algorithms for real-world problems, such as optimizing agricultural benefits, can be the subject of future research.

## Supplementary Information

Supplementary Information.

## Data Availability

The data analysed during this current study available from the corresponding author on reasonable request.
