# Supplementary material for: Neutrosophic goal programming technique with bio inspired algorithms for crop land allocation problem
Source: Sci Rep. 2024 Sep 16;14:21565. doi: 10.1038/s41598-024-69487-0 (PMC11405532; doi:10.1038/s41598-024-69487-0)
Supplement: Supplementary file 1 — Supplementary Information. [file 41598_2024_69487_MOESM1_ESM.docx]

**Supplementary material**

**Production, Profit, Expenditure and Labour per acre**

| Crop | Production | profit | Labour cost | Seed Cost | Pest & Fert cost | Miscellaneous  cost | Labour |
| --- | --- | --- | --- | --- | --- | --- | --- |
| Paddy ($y_{1}$) | 3430 | 73745 | 9150 | 3175 | 3310 | 3915 | 30.5 |
| Groundnut ($y_{2}$) | 1555 | 98742.5 | 5550 | 4500 | 2500 | 4000 | 18.5 |
| Cotton ($y_{3}$) | 1275 | 74268.75 | 19650 | 1500 | 5000 | 3400 | 65.5 |
| Paddy ($y_{4}$) | 3760 | 80840 | 9150 | 3175 | 3310 | 3915 | 30.5 |
| Groundnut ($y_{5}$) | 1455 | 92392.5 | 5550 | 4500 | 2500 | 4000 | 18.5 |
| Pearl Millet ($y_{6}$) | 1365 | 29347.5 | 4650 | 350 | 1180 | 1150 | 15.5 |
| Sweet Corn ($y_{7}$) | 2730 | 50505 | 6150 | 2000 | 2800 | 1100 | 20.5 |
| Paddy ($y_{8}$) | 2830 | 60845 | 9150 | 3175 | 3310 | 3915 | 30.5 |
| Brinjal ($y_{9}$) | 11600 | 232000 | 8850 | 1600 | 5500 | 3600 | 29.5 |
| Onion ($y_{10}$) | 9650 | 154400 | 10650 | 6000 | 5000 | 1900 | 35.5 |
| Sesame ($y_{11}$) | 355 | 24335.25 | 3900 | 650 | 1380 | 2620 | 13 |
